# A KPI-Based Model for Improving Aluminium Alloy Casting Processes: Effects on Material Properties of Castings

**DOI:** 10.3390/ma19132872

**Published:** 2026-07-05

**Authors:** Andrzej Pacana, Karolina Czerwińska

**Affiliations:** Faculty of Mechanical Engineering and Aeronautics, Rzeszow University of Technology, Al. Powstancow Warszawy 12, 35-959 Rzeszow, Poland; k.czerwinska@prz.edu.pl

**Keywords:** microstructural analysis, key performance indicator, KPI dashboards, process optimisation, production engineering, high-pressure die-casting (HPDC)

## Abstract

In the context of increasing requirements for product quality and reduction in environmental impact, the improvement of aluminium alloy casting processes has become an important research area within Industry 4.0 and industrial digitalisation. The aim of this study was to develop a KPI-based model supporting the optimisation of high-pressure aluminium alloy casting processes, considering its influence on material properties and environmental performance. An important aspect of the proposed model is its adaptive potential and its implementation in real industrial conditions, which increases its application relevance in modern production systems. A set of key performance indicators describing process stability, quality, and resource efficiency was defined and linked to casting process parameters. KPI dashboards were developed to support the visualisation and analysis of key process parameters in relation to main and auxiliary processes. The analysis included microstructure evaluation and mechanical property assessment as a function of KPI values. The results confirm that the integration of KPIs within an Industry 4.0-oriented approach under conditions of industrial digitalisation supports effective optimisation of high-pressure aluminium alloy casting processes, leading to improved material quality and improved production sustainability.

## 1. Introduction

Modern manufacturing companies operate in an environment characterised by dynamic technological change and increasing demands regarding efficiency, product quality and minimising environmental impact. The development of the Industry 4.0 concept is driving the digitalisation of production processes, the integration of monitoring systems and the use of data in the management of technological processes. This transformation involves the implementation of smart manufacturing systems, automation and analytical tools that support real-time decision-making [1,2]. The ability of companies to respond quickly to changes in production conditions and to identify factors affecting process stability is also becoming increasingly important [3]. At the same time, the integration of quality, economic and environmental aspects is playing an increasingly important role, aimed at minimising material losses, ensuring the efficient use of resources and reducing the energy intensity of processes [4,5]. These measures stem both from the need to maintain the competitiveness of enterprises and from the pursuit of sustainable development objectives and environmental requirements [6,7,8].

These development trends are of particular significance in the foundry industry [9], which is characterised by high technological complexity and considerable sensitivity to fluctuations in production parameters [10,11,12]. Foundries operate under conditions of increasing demands regarding the quality of castings, production repeatability and the reduction in material and energy costs [13,14,15]. At the same time, foundry processes are among the most energy- and material-intensive sectors of industry, which necessitates measures to improve environmental performance [16,17,18]. In response to these challenges, the development of modern foundry systems focuses on the integration of digital tools, monitoring systems and data analysis methods that support the identification of areas requiring improvement and the maintenance of process stability [19,20,21,22].

The casting of aluminium alloys is one of the key areas of the modern metal industry, mainly due to the widespread use of these materials in sectors requiring high strength whilst minimising the weight of structures, such as the aerospace, automotive and energy sectors [23,24,25]. This process is characterised by a high degree of complexity arising from the interdependence between process parameters and the formation of the material’s microstructure, which directly influences the mechanical and functional properties of the castings [26]. Consequently, even slight deviations in process parameters can lead to significant changes in quality, which underlines the importance of process stability and effective control [27].

High-pressure die-casting (HPDC) of aluminium alloys is of particular importance in the foundry industry; thanks to its high productivity and the ability to produce components with complex geometries, it is one of the most widely used casting technologies [28,29,30]. However, this process is highly sensitive to variations in process parameters, such as the temperature of the molten metal and the mould, injection speed and pressure, mould cavity filling conditions, solidification time and heat dissipation [31,32,33,34]. Uncontrolled deviations in these parameters can lead to casting defects, including porosity, microcracks and structural inhomogeneities, which result in reduced reliability and durability of the products [35,36,37,38].

In the analysis and optimisation of the HPDC process, the material properties of the castings—which are determined by the crystallisation process and the thermal and hydrodynamic conditions prevailing during mould filling—are of key importance [34,35]. Controlled microstructure formation is fundamental to achieving the required mechanical properties, such as tensile strength, hardness and fatigue resistance [39,40]. Increasingly, these analyses also encompass environmental aspects related to energy consumption, the efficiency of raw material utilisation and the level of production losses, which are directly linked to the quality and stability of the process [41,42,43,44]. This approach is in line with the concept of sustainable production, combining the optimisation of material properties with a reduction in the process’s environmental impact [45,46,47].

The high-pressure die-casting process for aluminium alloys is characterised by a complex structure comprising both operations directly related to the production of the casting and activities that support the stability and repeatability of the process. The individual stages differ in terms of their impact on product quality, material properties, material wastage and energy efficiency. For this reason, it is important to distinguish between the main processes, which are responsible for shaping the casting and its functional properties, and the auxiliary processes, which ensure appropriate technological and organisational conditions. This division facilitates the identification of areas affecting process stability, casting quality and the environmental efficiency of production. Table 1 presents the structure of the HPDC process, taking into account the key technological stages and their significance for casting quality and the efficiency of the production process.

The process stages listed in Table 1 demonstrate that HPDC of aluminium alloys is a multistage process in which both primary and auxiliary operations influence the quality of the castings, the stability of the process, and the efficient use of resources. Operations directly involved in casting are of key importance to the quality of the process, as they determine the course of phenomena that affect the material structure, the presence of casting defects, and the final mechanical properties of the products. Activities supporting the technological process also play an important role; their purpose is to ensure stable production conditions, efficient utilisation of resources, and a reduction in the environmental impact.

In the case of large-scale die-castings, such as rear floor panels for cars produced using HPDC technology, the concept of cooling time requires a more detailed definition than is the case for conventional small-scale die-castings. This parameter should be understood as the time required to dissipate heat from the casting and for it to reach a temperature that ensures the mould can be safely opened and the casting removed without the risk of deformation, cracking or a loss of dimensional stability. Due to the considerable size of structural castings and the varying thickness of their walls, the cooling process proceeds unevenly in different areas of the mould cavity. Consequently, the mould temperature should not be analysed as a single average value but as a temperature distribution recorded at multiple measurement points. In industrial practice, it is particularly important to monitor the temperature in areas with the highest concentration of material, at the junctions between stiffening ribs and load-bearing elements, in the regions where structural components are fixed, near the gating system, and at the points furthest from the metal feed points. These locations are characterised by differing heat transfer and solidification conditions, which can lead to microstructural inhomogeneities, porosity or deformation of the casting. Monitoring the cooling time and mould temperature in critical zones provides valuable data for assessing process stability.

In the analysed process of HPDC of aluminium alloys, management processes can also be identified, which are related, among other things, to production planning, organisational oversight, and the analysis of production data. This group of processes plays an important role in the company’s operations and supports the implementation of Industry 4.0 objectives. However, within the scope of this study, these processes were not considered as a separate area of analysis because their impact on the material properties of castings and the environmental efficiency of the process is indirect. The main and auxiliary processes directly related to the HPDC process are largely responsible for the quality of the microstructure, the presence of casting defects, energy consumption, and material losses.

The rapid development of digital solutions in foundry technologies is one of the key outcomes of the implementation of the Industry 4.0 concept [48,49,50]. This involves the increasingly widespread use of cyber-physical systems that integrate the production area with the company’s IT infrastructure [51]. The integration of monitoring systems, industrial sensors and real-time data analysis tools enhances the ability to monitor technological parameters and supports a better understanding of the HPDC process [52,53,54]. This enables not only the recording of process data but also their predictive analysis, which helps to reduce production variability [55,56]. Of particular importance is the identification of relationships between technological parameters and the quality of castings, including the occurrence of nonconformities and the formation of material properties [57,58]. The use of advanced data analysis methods supports the identification of critical process points and parameters that have the greatest impact on the quality stability of production, forming the basis for its optimisation [59,60].

A review of the relevant literature indicates that the improvement of the HPDC process is one of the main areas of research in modern foundry engineering. Table 2 presents a selection of publications on the improvement of the HPDC process, together with a description of their main research focus.

Based on the literature listed in Table 2 on the field of HPDC, three main research areas can be identified. The first concerns the influence of the process parameters on the quality of the casting [40,61,62,63,64]. The second covers the analysis of phenomena occurring at individual stages of the process, such as mould filling, solidification, and heat transfer, taking into account issues such as flowability (EFL), the IHTC coefficient, and the mechanisms of structure and defect formation [65]. The third strand involves modelling and simulation of the entire process using CAE tools, which integrate thermal cycles, the injection stage, and solidification, and support the optimisation of process conditions based on numerical and experimental data [43,66,67].

Additionally, there is a further research area related to the digitalisation and intelligent monitoring of the HPDC process, encompassing the use of machine learning, predictive models, and real-time monitoring systems. These solutions support the analysis of process conditions and the improvement of casting quality, aligning with the principles of Industry 4.0 [68,69].

The literature review indicates a gradual shift from the analysis of individual parameters and local physical phenomena towards integrated, digital methods for managing and optimising the HPDC process, within the Industry 4.0 paradigm.

The ongoing digitalisation of HPDC processes and the development of analytical tools have increased the importance of quantitative process performance evaluation. In this context, key performance indicators (KPIs) play a central role in assessing process quality, stability, and efficiency from a multidimensional perspective [70,71]. The integration of data-driven approaches with the Industry 4.0 concept requires continuous process monitoring supported by measurable indicators [72]. The implementation of KPIs in foundry environments facilitates the development of structured evaluation systems, enabling performance comparison under different production conditions and the identification of factors affecting process stability and repeatability [73]. Appropriately selected KPIs also support the standardisation of production performance assessment in complex casting processes involving numerous interrelated technological parameters [74,75]. Furthermore, continuous KPI analysis supports process optimisation and informed decision-making regarding technological parameters and production organisation [76].

The importance of KPI systems is also increasing in the integration of technological data with production and environmental management systems. KPIs are becoming an integral part of digital decision-support systems, facilitating process performance analysis, assessment of technological changes, and evaluation of resource-use efficiency [77,78]. Their implementation supports the development of integrated casting process management models that simultaneously address quality, technological, and environmental aspects [79].

In view of the above, the aim of this study was to develop a model for the use of key performance indicators (KPIs) in the analysis and improvement of the HPDC process for aluminium alloys in the context of industrial digitalisation.

The originality of this study stems from its comprehensive approach to the analysis and improvement of the high-pressure die-casting process for aluminium alloys, integrating technological, material, quality, and environmental aspects within a single KPI-based analytical model. In the approach presented, key performance indicators were not limited solely to monitoring the casting process but were treated as a tool to aid in identifying the relationship between the course of the technological process and the material properties of the castings. A particular importance in the developed HPDC process improvement model was attributed to linking process data with the analysis of porosity, microstructure and mechanical properties of aluminium castings, which represents a development of classical approaches focussing mainly on the evaluation of the process’s operational efficiency.

Another novel aspect of this study lies in the extension of the KPI analysis to include environmental factors related to the process’s energy consumption, material efficiency, and reduction in production losses. Consequently, the proposed model for improving the HPDC process is multicriteria in nature, taking into account quality, technological, material and environmental requirements simultaneously. The approach presented is in line with the principles of sustainable development and smart manufacturing systems characteristic of Industry 4.0. An important component of the study is also the integration of process data with analytical tools that support the ongoing evaluation of process stability and the identification of technological parameters that affect production quality variability.

The practical value of the developed model is highlighted by the creation of sample KPI dashboards dedicated to the HPDC process. The dashboards integrate technological, quality, and environmental data within an integrated production process analysis system. The proposed solution expands the traditional approach to process monitoring by shifting from the passive recording of technological parameters to actively supporting the improvement of the casting process and identifying avenues for optimising and refining HPDC technology. Another important aspect of the developed model is its adaptability and the possibility of implementing it in real-world industrial conditions, which enhances its practical significance within modern production systems.

## 2. Research Methodology

The study focused on the evaluation and improvement of high-pressure die-casting (HPDC) processes for aluminium alloys through the use of key performance indicators (KPIs) integrated with Industry 4.0 solutions. The analysis encompassed technological aspects of the process as well as their relationships with casting quality, material properties, and environmental performance.

A systems approach was adopted, in which the HPDC process was assessed using a set of interrelated KPIs representing different areas of production. Particular attention was given to the relationships between process parameters, quality outcomes, and the potential for their monitoring and optimisation in a digital environment.

**Figure 1:**
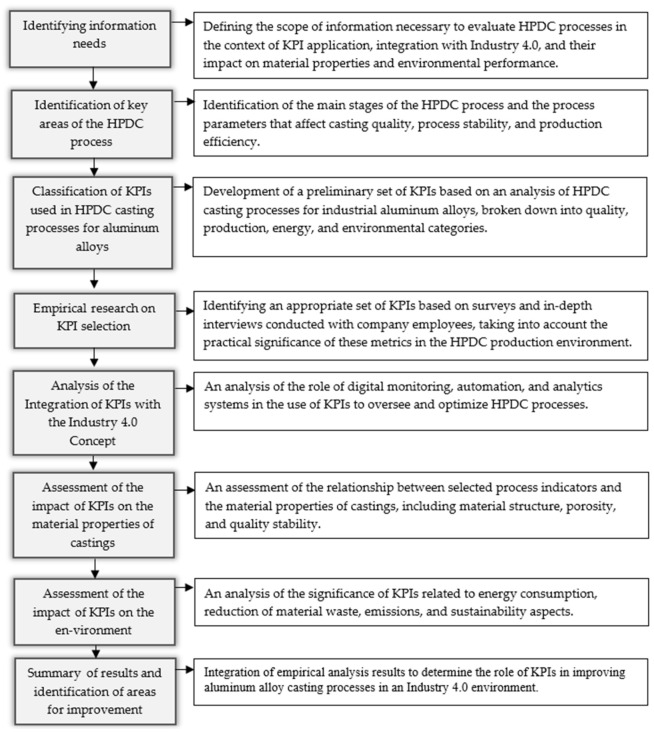
General flowchart for evaluating and improving HPDC processes using KPIs.

The adopted research methodology is presented in Figure 1 as a conceptual model illustrating the successive stages of the analysis and integrating the key elements discussed in the following sections.

The HPDC process evaluation and improvement model presented in Figure 1 illustrates the sequence of research stages related to the identification, structuring, verification, and integration of KPIs within an Industry 4.0 environment. The process began with the identification of information needs by defining the data required to assess casting quality, process stability, material properties, and environmental performance.

Subsequently, the HPDC process was decomposed into its key technological stages, including alloy preparation, injection, solidification, and finishing operations, together with the assignment of process parameters relevant to quality control and production stability. Based on this analysis, a preliminary set of KPIs was developed for both main and auxiliary processes.

In the next stage, a literature review was conducted to identify a suitable set of KPIs for the aluminium alloy casting process. Based on the findings, the method proposed by Oliveira et al. [80], which incorporates employee input into KPI selection, was adopted. Accordingly, questionnaires and direct interviews were conducted with employees experienced in HPDC process implementation, process monitoring, material property evaluation, and environmental and energy performance assessment.

The adopted methodology provided a practical framework for KPI identification and evaluation in an industrial environment. Data were collected from employees representing various areas related to die-casting operations and Industry 4.0 implementation, including production and foundry managers, process technologists and engineers, quality and materials specialists, maintenance and automation engineers, as well as personnel responsible for energy management, environmental protection, production data analysis, and KPI monitoring. Respondents were selected in cooperation with the company’s Managing Director and Human Resources Manager based on their organisational roles and competencies.

Face-to-face interviews with employees who possess expert knowledge in the organisation and supervision of the high-pressure die-casting process, quality management, energy efficiency, and implementation of Industry 4.0 solutions within the company were conducted. Respondents held positions related to the analysis of process parameters, assessment of the material properties, and the improvement of the environmental and production efficiency of the HPDC process. This approach facilitated the collection of a diverse range of opinions.

The questionnaire used in the study was designed to enable a comprehensive assessment of the suitability of the identified indicators in relation to the aluminium alloy casting process, taking into account: the principles of Industry 4.0, the material properties of the castings, and the environmental performance of the HPDC process. The questionnaire was structured into four main sections.

The questionnaire consisted of five sections. Section 1 introduced the purpose of the study and provided instructions for completing the survey, including the assessment scale, where 1 indicated very low importance and 5 indicated very high importance of a given KPI. Section 2 collected respondents’ profile information, such as their area of responsibility, position, work experience, education level, and experience with foundry processes and digital tools.

Section 3 and Section 4 focused on evaluating KPIs related to support processes and the main stages of the HPDC process, respectively. To ensure consistent assessments, each KPI was accompanied by its name, definition, calculation method, and a brief explanation of its relevance to process improvement. Section 5 contained open-ended questions, allowing respondents to provide additional comments, propose new KPIs, and identify opportunities for improving the HPDC process through Industry 4.0 solutions.

Based on the responses received, the indicators under analysis were evaluated and classified. The KPI selection process consisted of two stages. In the first stage, the survey results were analysed, and KPIs with high average ratings and little variation in the responses of the respondents were identified. KPIs for which the average respondent rating was at least 3.5 were selected for further analysis. In the second stage, the initially selected indicators were validated by company experts in terms of their usefulness in monitoring the HPDC process, the material properties of the castings, and energy and environmental efficiency.

This approach led to the creation of a final set of KPIs of significant theoretical and practical importance, while simultaneously limiting the influence of subjective assessments.

A total of 62 respondents participated in the study, and 61 correctly completed questionnaires were included in the subsequent analysis. The high response rate should be considered satisfactory, which attests to the reliability and quality of the collected data. The number of study participants resulted from the adopted methodological assumption, according to which the questionnaire was directed exclusively at management staff and individuals who hold decision-making and supervisory roles in areas related to the aluminium alloy casting process and the implementation of Industry 4.0 solutions.

The respondents who participated in the survey mainly represented management (57%) and technical specialists and process engineers (43%) involved in the execution and supervision of the aluminium alloy die-casting process and the implementation of Industry 4.0 solutions. Most of the respondents (76%) had a technical or engineering background, while the average service at the company was 8 years. The respondents represented various areas of the company’s operations, namely aluminium alloy melting and preparation (21%), the HPDC die-casting process (34%), quality control of castings and evaluation of material properties (18%), maintenance (12%) and areas related to energy management, environmental protection, and KPI analysis (15%).

To supplement and expand upon the results of the survey, individual face-to-face interviews were conducted with management representatives and specialists involved in the implementation and supervision of the aluminium alloy die-casting process. The main objective of the face-to-face interviews was to obtain detailed information regarding the significance and potential applications of KPIs in monitoring the HPDC process, evaluating the material properties of castings, analysing environmental performance, and supporting digital process monitoring in accordance with the Industry 4.0 concept. The interviews were conducted based on a preprepared interview script, developed in accordance with the thematic scope of the research.

A semi-structured interview format was adopted, which, in addition to a set of preprepared questions, allowed for the exploration of additional topics that arose during the conversation. This approach facilitates the collection of detailed opinions from respondents on the practical aspects of monitoring process parameters, the use of production data, and the evaluation of the usefulness of specific KPIs in an industrial environment.

The structure of the face-to-face interview was developed based on the layout of the survey questionnaire. Section 1 specified the purpose, scope of the conversation, and principles of anonymity and voluntary participation. Section 2 covered information on the respondents’ job positions, scope of duties, and professional experience in the area of casting processes and production monitoring systems.

The main part of the interview focused on evaluating indicators related to both the auxiliary processes and the main HPDC process. Attention was drawn to issues concerning the impact of technological parameters on the quality and material properties of castings, the ability to monitor the energy and environmental efficiency of the process, and the potential for using digital tools to collect and analyse process data. The surveyed employees also identified barriers related to the implementation and practical use of KPIs in a manufacturing company.

The combination of survey research and direct interviews helps to obtain a complete picture of the analysed issue through the use of quantitative and qualitative data, which are important in the process of identifying and evaluating KPIs. This approach contributes to the identification of the conditions of the aluminium alloy casting process and the assessment of the importance of indicators related to material properties and the environmental efficiency of the HPDC process.

In the next stage, the developed model calls for an analysis of the integration of the selected set of KPIs with Industry 4.0 tools, particularly real-time process data monitoring systems, control automation solutions, and data analytics tools that support process parameters.

The subsequent phase of the study included an analysis of the relationship between KPIs and the material properties of castings, including an assessment of the impact of process parameters on the microcrystalline structure, porosity, and quality stability of the products, as well as an analysis of their links to the environmental performance of the process, including energy consumption, material consumption, and emissions.

The final stage of the model involved synthesising the results of the literature and empirical research to identify the most critical KPIs and developing visual dashboards. The dashboards were designed using Microsoft Power BI (Business Intelligence) Desktop 2.154.1260.0, a tool employed for data analysis and the creation of interactive visualisations and reports. The developed method for visualising the KPI results enables detailed and rapid analysis and interpretation of data. This approach significantly helps identify ways to improve HPDC processes in the context of the digital transformation.

## 3. Results and Analysis

### 3.1. Classification of KPIs in the HPDC Process, Taking into Account the Quality, Microstructure, and Properties of Aluminium Alloys

The accuracy and usability of the developed model were verified at a company operating in the aluminium industry. The company in question has been in business for over two decades and is located in southeastern Poland. The company’s core business is the production of aluminium alloy products used, among others, in the automotive, aerospace, energy, railway, and robotics industries, as well as in the fields of mechanical engineering and engine technology. The research was carried out in the second and third quarters of 2025.

Based on the process classification and a review of the relevant literature, a preliminary set of potential KPIs was developed. Subsequently, two working meetings were held with representatives of the company’s management, including managers of individual organisational units and their deputies responsible for achieving operational goals, supervising teams, and ensuring the company’s performance. The key objective of the meetings was to identify available data on the quality of aluminium alloy castings, with particular emphasis on the parameters that influence the formation of the microstructure and the performance properties of the material. The analysis covered information regarding, among other things, the occurrence of casting nonconformities, the stability of process parameters, the internal structure of castings, and the mechanical properties of the resulting products. Additionally, information was gathered on improvement measures aimed at enhancing the metallurgical quality of alloys, reducing casting defects, and increasing the repeatability and reliability of the high-pressure die-casting process. During the discussion, issues related to the level of implementation of Industry 4.0 principles in the analysed company were also addressed.

On the basis of an analysis of the main stages of the high-pressure die-casting process for aluminium alloys and on the results of the surveys conducted, a set of key performance indicators (KPIs) was developed to assist in monitoring the process stability, metallurgical quality, and parameters affecting the microstructure and performance properties of castings. Table 3 presents the proposed set of KPIs along with their mathematical form, variable notation, and unit of measurement.

The KPIs presented in Table 3 form the basis for a quantitative assessment of key parameters in the HPDC of aluminium alloys. The mathematical relationships presented enable monitoring of the stability of successive stages in the manufacturing process and allow for an assessment of the variability of parameters that affect the metallurgical quality and repeatability of the castings produced. The presentation of calculation formulas, variable designations, and units of measurement contributes to standardising the method of process data analysis and supports the implementation of KPIs within systems for monitoring and improving production processes.

It should be emphasised that, in the case of microstructure-related parameters (Table 3), such as the average SDAS (Secondary Dendrite Arm Spacing) value, the proposed model does not involve direct metallographic observation inside the mould during the solidification process. This is due to the conditions characteristic of the high-pressure die-casting of aluminium alloys, including the presence of release agents, oil mist and intensive mould cooling, which preclude reliable in situ optical detection. In the proposed concept, data on the cooling profile, recorded by sensors integrated into the mould, are used to numerically estimate the cooling rate and, subsequently, the SDAS value. To verify the accuracy of the estimates, samples taken from a suitably designed process component (e.g., a sprue) undergo automatic cleaning and image analysis using digital microscopy at an automated measurement station located outside the working area of the HPDC machine. This architecture combines process data with the results of material testing, in line with the principles of Industry 4.0 and the digital monitoring of KPIs.

Due to the microstructural gradient characteristic of HPDC castings, the SDAS analysis is carried out within a defined region of interest (ROI), located in the central zone of the cross-section of the component under investigation. The selection of this zone helps to minimise the influence of local variations in the thickness of the fine-grained surface layer and ensures that the results are representative for the assessment of solidification process stability. The fixed location of the measurement area and a standardised sample preparation procedure ensure a high degree of comparability of results between successive production batches and minimise the impact of geometric variations in the castings on the assessment of the SDAS index.

However, the mathematical form of the KPIs alone does not fully reflect their technological and quality significance. Therefore, Table 4 presents an expanded description of the proposed KPIs. This approach supports a better alignment of the indicators analysed with the practical aspects of the HPDC process and facilitates the identification of areas that require improvement measures.

The summary presented in Table 4 allows for the correlation of individual indicators with the objectives pursued during the main stages of the high-pressure die-casting process for aluminium alloys. The proposed KPIs are not only related to monitoring the stability of process parameters but also serve to evaluate factors influencing the metallurgical quality, microstructure formation, and performance properties of castings.

Interpreting individual KPIs in this context highlights their importance not only as control measures but also as tools for describing process stability and its impact on material structure formation. An analysis of the objectives of the proposed KPIs indicates that effective monitoring of the HPDC process requires simultaneous consideration of thermal, flow, and quality parameters. KPIs related to porosity control, injection process stability, and solidification behaviour are particularly important. These parameters have a direct impact on the uniformity of the material structure and the mechanical properties of the products.

The summary of KPIs (Table 3 and Table 4) serves as the basis for further analysis of the auxiliary processes. Although these processes do not directly contribute to the formation of the casting, their stability and proper execution have a significant impact on the conditions of the HPDC process, the metallurgical quality of the alloy, and the properties of the reproducibility of the final product. Expanding the scope of the analysis will allow for the examination of the entire production system, taking into account both processes that directly affect casting quality (main processes) and those that support its execution (auxiliary processes), thereby ensuring a comprehensive evaluation of the HPDC system under study.

Table 5 presents selected KPIs for auxiliary processes along with their mathematical form and variable notation. The included indicators are related to issues related to the stability of chemical composition, control of thermal conditions, mould surface preparation, and the use of secondary material in the production process.

The KPIs for the auxiliary processes presented in Table 5 indicate that the processes supporting the implementation of HPDC technology are crucial to ensuring the stability of process conditions and the metallurgical quality of aluminium alloys. Despite the indirect nature of the impact of this group of processes, nonconformities occurring at stages such as charge preparation, molten metal transport, or mould preparation can lead to disruptions in the process flow and a deterioration in the quality of the final castings.

The stage of controlling the chemical composition stability of the charge is particularly important, as variations in the content of alloying elements affect the solidification process, the proportion of intermetallic phases, and the mechanical properties of the products. Equally important is minimising heat loss during the transport of molten metal, as an excessive drop in temperature can lead to poor mould filling conditions and an increased risk of casting defects.

Proper preparation of the mould surface is also crucial, as it affects local heat transfer conditions, the stability of the solidification process, and the surface quality of the castings. With regard to scrap material, it is important to maintain a balance between the efficiency of material utilisation and the preservation of the required metallurgical quality of the alloy. An excessive proportion of recycled material can lead to higher levels of contamination and greater variability in the microstructure of the castings.

Table 6 presents a detailed interpretation of the KPIs developed in relation to auxiliary processes, including the objectives of individual indicators and their significance in the context of the quality, microstructure and properties of aluminium alloys.

The indicators presented in Table 6 highlight the importance of auxiliary processes in maintaining stable conditions for HPDC technology. An analysis of the objectives of the developed KPIs and their impact on the quality, microstructure and properties of aluminium alloys indicates that these processes influence many phenomena that occur during the melting, transport, and solidification of the molten metal.

Particularly important are measures related to controlling the homogeneity of the feedstock, minimising heat loss, and ensuring stable mould operating conditions. Problems occurring at these stages can result in changes in crystallisation conditions, increased porosity, structural inhomogeneity, and deterioration in the mechanical properties of cast products. The significance of auxiliary processes is also evident in the use of return material, of which the quantity should be controlled with regard to the metallurgical quality of the alloy.

Incorporating auxiliary processes into the KPI framework enhances the representation of the relationships that occur in HPDC of aluminium alloys and broadens the scope of process stability assessment beyond activities directly related to casting formation.

The set of key performance indicators (KPIs) developed goes beyond the function of monitoring process parameters on an ongoing basis. Linking the KPIs to the individual stages of the HPDC process and relating them to the quality of the castings, microstructure and material properties forms the basis for supporting technological decision-making. An analysis of changes in the values of these indicators can provide grounds for adjusting process parameters—such as molten metal temperature, pouring conditions, intensification pressure or the solidification profile—even before any quality nonconformities occur.

### 3.2. Integrating KPIs with the Industry 4.0 Concept in the HPDC Process

The integration of KPIs with solutions specific to the Industry 4.0 environment extends their application beyond traditional process monitoring. Data obtained from measurement systems, process sensors and material testing can be analysed continuously, creating a coherent environment that supports the assessment of process stability, the identification of technological deviations and the analysis of relationships between process parameters, microstructure and the properties of castings.

The proposed KPIs (Table 3 and Table 5) relate to technological and material parameters that are significant in the context of the metallurgical quality of aluminium castings, as well as the mechanisms governing the formation of their microstructure and performance properties. The stability of the HPDC process parameters influences the course of the phenomena occurring during mould filling and alloy solidification, affecting, among other things, the porosity level, structural homogeneity and mechanical properties of the cast products. In industrial practice, effective monitoring and analysis of these relationships require the use of modern tools that enable the recording, processing, and ultimately interpretation of process data in real time.

Therefore, it is appropriate to relate the developed set of KPIs to the principles of the Industry 4.0 concept, which is based on digitisation of production processes, data integration, and the use of intelligent process monitoring systems. The implementation of industry-4.0-compatible solutions enables more effective monitoring of key HPDC process parameters, faster identification of technological deviations, and supports the making of accurate corrective decisions related to maintaining the quality stability of castings.

Table 7 presents the relationship between the set of KPIs for the HPDC of aluminium alloys and representative solutions and tools that align with the principles of Industry 4.0.

The summary presented in Table 7 indicates that the concept of Industry 4.0 can be applied to every stage of the HPDC process. Integration of digital systems, process sensors, automatic data acquisition systems, and analytical tools enables real-time monitoring of process parameters and the efficient identification of process deviations. Solutions that allow real-time (or near-real-time) monitoring of parameters are of significant importance, as the stability of the HPDC process has a direct impact on the metallurgical quality, microstructure, and performance properties of castings.

An analysis of the relationships presented in Table 7 indicates that modern Industry 4.0 systems are not limited solely to production automation but also encompass areas related to quality analysis, material traceability, process data archiving, and the prediction of quality nonconformities. This enables more effective monitoring of factors that influence the tightness, mechanical strength, fatigue resistance, and structural uniformity of aluminium castings.

The use of process data monitoring and analysis systems helps to improve the environmental efficiency of the HPDC process. Continuous monitoring of material losses, the proportion of recycled material, and energy consumption helps reduce the number of defects generated and reduce the consumption of virgin raw materials. In this context, it is crucial to monitor the stability of the melt and liquid metal transport parameters, as well as the efficiency of recycled material, because these factors affect both the quality of the alloy and the energy consumption of the process.

The information in Table 7 also underscores the importance of integrating process data from various stages of the process. Integration of MES, SCADA, and ERP systems, as well as quality monitoring tools, forms the foundation for building a cohesive digital environment that supports informed supervision and improvement of the HPDC process. This approach promotes increased production repeatability, improved material utilisation, reduced scrap rates, and the stabilisation of the mechanical and quality properties of aluminium alloy castings, while simultaneously supporting the principles of sustainable development and the circular economy.

An important aspect of integrating the relationships presented (Table 7) is their display using operator dashboards, which serve as a layer for visualising production data in an Industry 4.0 environment. These dashboards enable the monitoring of KPIs for each stage of the HPDC process, while simultaneously referencing current process parameter values and signals from automation systems and databases. As a result, operators and engineering personnel receive clear and condensed information about the process status, which significantly aids in making appropriate decisions in real time.

An integrated environment based on SCADA and MES systems, as well as business intelligence tools, was used to monitor and analyse the developed set of KPIs in the HPDC of aluminium alloys. The SCADA layer performs real-time data acquisition from industrial automation devices (including liquid metal and mould temperature sensors, injection system pressure sensors, alloy dosing systems, and vision-based quality control systems).

The collected data are sent to the MES system, where they are aggregated, validated, and assigned to a production context, that is, a batch of materials, a machine, or a shift. At this stage of the analysis, quality and material information is integrated, including laboratory test results and microstructure and material consumption data. This integration enables the determination of KPIs that provide insights into process stability, casting quality, and material efficiency.

The processed data are ultimately visualised in a BI environment, where they are presented in the form of dynamic indicators, charts, and trends. The use of this architecture facilitates the transformation of operational data into measurable and relevant KPIs, which supports continuous monitoring of the HPDC process, the identification of nonconformities, and the assessment of the impact of individual process parameters on material quality and the environmental efficiency of production.

**Figure 2:**
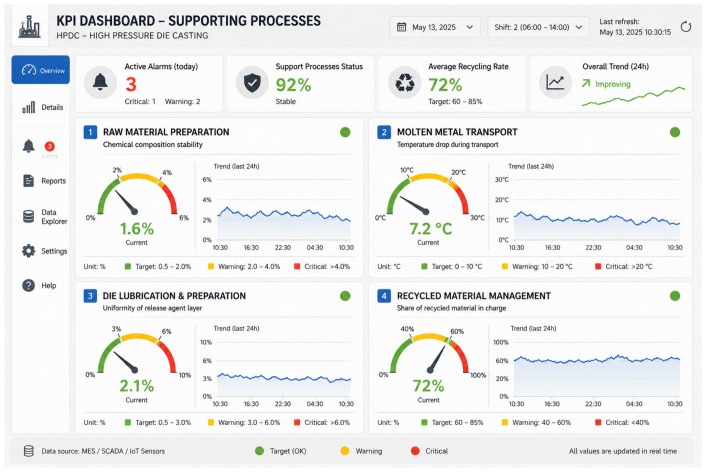
Sample KPI dashboard for support processes in HPDC of aluminium alloys.

Figure 2 shows an example KPI dashboard developed for an aluminium processing company, related to auxiliary processes carried out using high-pressure die-casting (HPDC) technology.

The KPI dashboard relating to auxiliary processes (Figure 2) focuses on monitoring factors affecting the stability of process conditions, such as charge preparation, molten metal transport, mould preparation and the recycling of return material. It has been designed as a multi-level visualisation dashboard enabling real-time monitoring of key performance indicators that help to reduce process variability, improve material efficiency and maintain consistent production conditions. The upper section of the dashboard displays aggregated information on the status of auxiliary processes, including the number of active alarms, the overall status of monitored processes, the material recycling rate, and trends in KPI changes over the analysed period. The central part of the dashboard consists of modules corresponding to individual auxiliary processes, equipped with gauge-type indicators enabling a quick assessment of current KPI values against defined reference thresholds, as well as trend charts illustrating the variation of parameters over time. In addition, a uniform colour scheme based on traffic light logic (green–yellow–red) has been used, supporting the intuitive identification of KPI achievement levels and potential process deviations. This dashboard design ensures a clear presentation of data and supports the rapid implementation of corrective actions in an Industry 4.0 environment.

**Figure 3:**
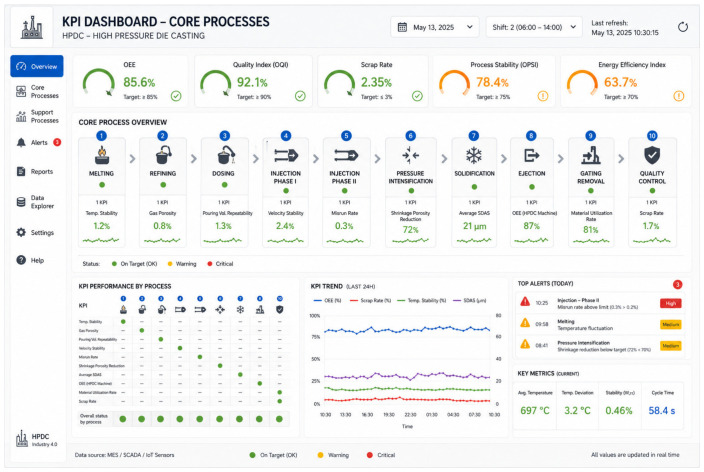
Sample KPI dashboard for key technological processes in HPDC of aluminium alloys.

The KPI dashboard shown in Figure 3 is related to the main technological processes carried out in HPDC of aluminium alloys. The developed visualisation system integrates key performance indicators (KPIs) associated with individual process stages.

The KPI dashboard relating to the main technological processes of HPDC of aluminium alloys (Figure 3) has been designed as a multi-level visualisation dashboard that integrates key performance indicators associated with the individual stages of the HPDC process. The upper section of the dashboard displays aggregated process performance indicators, including Overall Equipment Effectiveness (OEE), the Quality Index (OQI), the scrap rate, process stability (OPSI) and energy efficiency. The central part of the dashboard consists of a sequential visualisation of the main technological stages, including melting, refining, liquid metal dosing, both phases of the piston stroke, pressure intensification, solidification, casting removal, gating removal and quality control (numbers from 1 to 10). For each stage, the assigned KPI is displayed along with its current value and a summary assessment of the process status.

The lower section of the dashboard contains a matrix showing KPI performance across individual processes, trend charts for selected indicators, and an alerts panel displaying current deviations in process parameters. This is supplemented by key process parameters such as molten metal temperature, temperature deviation, process stability index and production cycle time. The use of a uniform colour scheme based on traffic light logic (green–yellow–red) facilitates the rapid identification of areas requiring intervention and the assessment of the extent to which target KPI values are being met. This dashboard design enables comprehensive monitoring of the HPDC process, supporting the analysis of the relationships between technological parameters, microstructure and the final quality of castings within an Industry 4.0 environment.

The developed KPI dashboards (Figure 2 and Figure 3) serve as an example of the practical application of the Industry 4.0 concept in the monitoring of HPDC processes for aluminium alloys.

The use of integrated visualisation dashboards supports real-time monitoring of KPI values, rapid identification of process deviations, and analysis of the relationship between technological parameters and casting quality. At the same time, dashboards form the basis for implementing intelligent production monitoring systems, trend analysis, and predictive improvement of HPDC processes within a digital production management environment.

The model presented here is not a proposal for a new high-pressure aluminium alloy casting technology, but rather a conceptual approach to managing the production process through the integration of technological parameters, key performance indicators, microstructure assessment and the material properties of castings. From an industrial perspective, this approach facilitates more informed technological decision-making, minimises process variability and reduces casting defects. At the same time, the integration of process and material data aligns with the principles of Industry 4.0, laying the foundations for the development of digital quality monitoring systems in HPDC processes.

## 4. Discussion

In the context of improving the HPDC process, several parallel lines of research can be identified from a review of the literature. These include both studies on the influence of process parameters on the quality of the casting [40,61,62,63,64], as well as analyses of phenomena that occur in specific stages of the process, such as mould filling, solidification, and heat transfer, together with a description of the associated physical mechanisms determining the structure and defects [65]. Another significant area is research on modelling and simulating the entire process using CAE tools, which integrate successive stages of the production cycle and support its optimisation based on numerical and experimental data [43,66,67]. Complementarily, a field related to digitalisation and intelligent process monitoring is developing, encompassing machine learning methods, predictive models, and real-time systems, which aligns with the principles of Industry 4.0 [68,69]. The reviewed literature indicates a gradual transition from the analysis of local parameters and physical phenomena to integrated, digital methods for managing and optimising the HPDC process.

An analysis of the use of key performance indicators (KPIs) in HPDC processes of aluminium alloys in the context of Industry 4.0 has shown that KPIs serve as a tool for measuring performance, but above all as a mechanism for controlling and stabilising the manufacturing process. The integration of KPIs with real-time monitoring systems enables dynamic management of process parameters, which directly translates to reduced production variability and a significant impact on the material properties of castings. Stabilising critical parameters, including the temperature of the molten metal, the injection pressure, and solidification conditions, enables a reduction in internal defects (such as shrinkage and gas porosity) and thus improves the uniformity of the structure and the reproducibility of the mechanical properties of the castings.

The findings confirm that key performance indicators should not only be viewed as a tool for assessing production efficiency but as a component of a real-time process control system. In particular, indicators related to energy consumption, scrap rates, or casting cycle times can play a predictive role, aiding in the early technological anomalies. Integrating KPI data with MES/SCADA systems enhances an organisation’s ability to make data-driven operational decisions (data-driven manufacturing).

In terms of environmental performance, the implementation of KPIs supports the informed management of energy and raw material consumption. Reducing the number of defects and optimising HPDC process parameters leads to a decrease in production scrap, which aligns with the concept of a circular economy. As a result, HPDC supported by Industry 4.0 systems becomes a more environmentally sustainable process.

Taking into account the technological, material and environmental aspects discussed, it was concluded that the implementation of a KPI system integrated with Industry 4.0 solutions leads to benefits at both the operational and strategic levels of the company. Of particular importance is the ability to improve the aluminium alloy casting process through continuous monitoring of technological parameters, which enhances process stability, casting quality, and resource efficiency. The long-term advantages of the model presented in this study include the following:

- Improving the stability and repeatability of the HPDC process;
- Reducing the variability of process parameters that affect casting quality;
- Improving control over parameters that affect the material properties of castings;
- Increasing the efficiency of energy and raw material utilisation;
- Reduction in the number of production defects, casting defects, and quality costs;
- Reduction in the amount of process scrap and material losses;
- Shorter response times to process parameter deviations thanks to real-time data analysis;
- The ability to implement predictive maintenance;
- Improving the long-term competitiveness;
- Improving organisational efficiency in the context of Industry 4.0.

Despite the numerous benefits of the proposed approach, the implementation of a KPI system integrated with Industry 4.0 solutions in HPDC processes also involves certain limitations and implementation challenges. Identifying these challenges is crucial for the practical application of the model in industrial settings and for further refinement of the developed solution. When conducting research based on the proposed model, the following implementation challenges were identified:

- The need to ensure high-quality and consistent process data;
- The complexity of integrating IT systems (MES, SCADA, and ERP) into the existing infrastructure and equipment in the company;
- The high costs of implementing and maintaining digital monitoring systems;
- The need for specialised staff capable of interpreting data and operating the systems;
- The risk of misinterpreting KPIs due to improper model calibration;
- Limited ability to directly transfer the model between companies with different technological profiles;
- The time-consuming nature of the implementation and organisational adaptation process;
- High requirements for digital infrastructure and data monitoring systems;
- Difficulties associated with synchronising data from different stages of the production process.

The effectiveness of implementing the proposed KPI system is largely dependent on the quality of the input data and the level of integration of the company’s IT systems. Inconsistencies in the data or incorrect interpretation of the indicators can lead to poor operational decisions. For this reason, not only is the implementation of technology important, but so is the appropriate organisational preparation and competence of the staff.

Integration of KPIs with the Industry 4.0 concept in HPDC processes is a key direction for the development of a modern foundry. It contributes to simultaneously increasing production efficiency, improving the material properties and structural quality of castings, and reducing the environmental impact of the process; however, it requires a systematic approach to data management and continuous improvement of the digital infrastructure.

The results presented include the identification of processes occurring in HPDC technology, the classification of key performance indicators, their correlation with casting quality, microstructure and material properties, and a concept for digital monitoring in line with the principles of Industry 4.0. The proposed model clarifies the relationships between the course of the technological process and the assessment of casting quality, providing a basis for further work on digital support for foundry process management.

## 5. Conclusions

High-pressure die-casting of aluminium alloys is currently one of the key manufacturing processes for products intended for the automotive, aerospace, and electrical engineering industries. Constantly increasing requirements with respect to material quality, process stability, and reduction in environmental impact mean that traditional methods of monitoring the casting process are no longer sufficient. In response to these challenges, the objective of this study was to develop a model for the use of key performance indicators (KPIs) in the analysis and improvement of HPDC of aluminium alloys in the context of industrial digitalisation. The focus was on the relationships between process parameters, the stability of operational indicators, and the material properties of the castings, including porosity, microstructure, and mechanical properties. Environmental aspects, such as energy and material consumption, were also taken into account. The approach was related to the concept of Industry 4.0, emphasising the role of process data integration and analytical tools in improving process stability and reducing quality variability.

This study focuses on the identification and selection of key performance indicators (KPIs) appropriate to the specific nature of the high-pressure die-casting (HPDC) process for aluminium alloys. Particular attention was paid to indicators describing process stability and the quality of the castings produced, taking into account characteristics such as porosity, microstructure and mechanical properties. The analysis was also extended to include environmental aspects related to energy and material consumption. The proposed approach was embedded within the Industry 4.0 framework, emphasising the importance of integrating process data and advanced analytical tools in improving process stability, reducing quality variability and supporting decision-making. To facilitate the practical implementation of the developed set of KPIs within an Industry 4.0 environment, dedicated management dashboards (KPI dashboards) were designed, enabling real-time monitoring and analysis of key HPDC process parameters.

The developed procedure model can serve as a valuable source of knowledge for representatives of the foundry industry, particularly companies using HPDC technology, as well as for specialists involved in quality management, production process optimisation, and the implementation of Industry 4.0 solutions. Future research will focus on the development of KPI monitoring systems to help reduce porosity and improve the quality stability of the casting process.

## Figures and Tables

**Table 1:** Stages of the high-pressure die-casting process for aluminium alloys, along with their characteristics.

Process Stage	Stage Description	Key Process Parameters	Potential Impact on Quality	Process Classification
Charging	Selection and preparation of aluminium alloy, alloying elements, and recycled material	Chemical composition, proportion of secondary material	Alloy chemical stability, material utilisation rate	Support
Alloy melting	Melting the charge in a melting furnace	Molten metal temperature (typically approx. 660–750 °C)	Energy consumption, metal oxidation	Primary
Purification and refining of molten metal	Removal of gases and non-metallic inclusions	Refining time, type of refining gas	Gas porosity, defect rate	Primary
Transport of molten metal	Transfer of the molten alloy to the machine chamber	Transport temperature, transport time	Temperature drop, process stability	Support
Lubrication and mould preparation	Application of lubricant and stabilisation of the mould temperature	Mould temperature, amount of lubricant	Surface quality, cycle time	Support
Filling the pressing chamber	Introduction of a specified amount of molten metal into the chamber	Metal volume, pouring time	Process repeatability	Primary
Phase I of piston movement	Slow movement of the metal toward the gating system	First-phase speed	Turbulence reduction	Primary
Phase II of piston movement	Rapid filling of the mould cavity	Second-phase speed	Underfill, entrapment of air	Primary
Pressure intensification	Additional pressure increase after the mould is filled	Intensification pressure	Reduction in shrinkage porosity	Primary
Solidification of the casting	Cooling and solidification of the alloy in the mould	Casting cooling time, mould temperature in critical zones	Microstructure, mechanical properties	Primary
Opening the mould and removing the casting	Separation of the casting from the mould	Cycle time	Productivity, OEE	Primary
Removal of the gating system	Removal of flash and feed channels	Processing method	Material losses	Primary
Quality control	Geometric, visual, and material evaluation of the casting	Number of defects, defect density	Scrap rate	Primary
Recycling of scrap material	Reuse of the gating system and scrap	Recycled material content	Sustainability KPI	Support

**Table 2:** Research papers related to the improvement of the HPDC process.

Area		Specifications	Author
HPDC process parameters	Research on three key parameters of die-casting	This study focused on the effect of HPDC process parameters (injection speed, vacuum, and intensification pressure) on the quality of aluminium castings. It was shown that intensification pressure and the use of a vacuum significantly reduce porosity and improve mechanical properties. It was confirmed that optimising the process parameters improves casting quality and reduces production losses.	Merchan et al. [40]
	Optimisation of HPDC process parameters using artificial intelligence	This research focused on the intelligent optimisation of the HPDC process using machine learning and multi-criteria optimisation. The XGBoost model and SHAP analysis were used to identify the impact of process parameters on quality, feed rate, and cycle time, followed by the NSGA-II algorithm to optimise production objectives. An improvement in casting quality and a reduction in material consumption and cycle time were demonstrated, confirming the effectiveness of AI methods in improving the HPDC process.	Wu et al. [61]
	The effect of process parameters on flowability and mould filling in HPDC	This research focused on the influence of HPDC process parameters on the flowability, defects, and mechanical properties of thin-walled aluminium castings. Numerical simulations and experimental verification of the mould filling process were used. A significant influence of casting conditions on mould filling and the uniformity of mechanical properties was demonstrated. The concept of effective flow length (EFL) was introduced as a measure of fluidity.	Niu et al. [62]
	Modelling and analysis of heat transfer in the HPDC process	This study focused on determining the heat transfer coefficient (IHTC) and heat flux in the HPDC process using experimental measurements and numerical simulations. Temperatures were recorded using thermocouples placed in the mould and casting, and IHTC values were calculated using the finite difference method and verified in ANSYS Fluent (version 1.53). An increase in injection pressure and velocity, as well as casting temperature, was shown to increase heat transfer intensity, whereas a higher mould temperature reduces it. The effect of vacuum application on the increase in IHTC was also confirmed.	Koru and Serce [63]
	Statistical optimisation of HPDC process parameters for casting quality	This research focused on optimising the HPDC process parameters to reduce porosity in aluminium castings. DOE methods and Taguchi analysis were used to evaluate the impact of key parameters, such as injection pressure, piston speed, alloy temperature, and cooling time. The cooling time and injection parameters have been shown to have a significant impact on the density and quality of the castings. Optimised process conditions reduced the defect rate by 61%.	Tariq et al. [64]
Stages of the HPDC process	Analysis of alignment solidification and microstructure formation in HPDC castings	This research focused on the solidification process of the aluminium alloy in the injection sleeve during CC-HPDC using numerical modelling. Metal flow, heat transfer, and the formation of ESC crystals were analysed, as well as the effect of piston movement on their distribution. It was demonstrated that ESC significantly influences the microstructure and mechanical properties of castings. The results may support the optimisation of HPDC process parameters.	Dou et al. [65]
The HPDC process	Improving the HPDC process by controlling vacuum conditions	This study investigated the effect of the ultrahigh vacuum-assisted HPDC process on the properties of large aluminium castings. It was demonstrated that a higher vacuum level improves yield strength, tensile strength, and ductility while reducing internal defects and improving microstructural uniformity. An increase in the resistance to crack initiation and propagation of cracks was also confirmed.	Yang et al. [66]
	Thermal management and digitally supported optimisation of the HPDC process in the context of Industry 4.0	This research focused on improving the efficiency of the HPDC process through the optimisation of die thermal management and a reduction in casting defects. It was indicated that improper mould cooling and the selection of process parameters affect casting quality and energy consumption. The use of conformal cooling channels manufactured using additive methods as a means of improving the thermal stability of the mould was discussed. The role of simulation, experimental design, and the ‘Smart Foundry’ concept in the context of Industry 4.0 and energy efficiency in foundry operations was explored.	Anand et al. [43]
	Modelling and optimisation of the HPDC process using CAE simulation	This research focused on simulating the HPDC process using CAE (ProCAST). The model included die thermal cycles, mould filling, and solidification of the Al-Si alloy, and heat transfer coefficients were calibrated based on experimental data. Based on this, the thermal cycles and injection profile were optimised, which reduced defects and improved the mechanical properties of the castings.	Dou et al. [67]
Digitisation of the HPDC process	Virtual sensors and predictive monitoring of HPDC process conditions	This study focused on the use of LSTM networks to predict the temperature and pressure conditions within the mould during the HPDC process. The model was trained on data from casting cycles and then used to forecast temperature and pressure as an alternative to physical sensors. High accuracy in temperature prediction and good accuracy for pressure were demonstrated, with a decrease in effectiveness over longer time horizons. The possibility of replacing selected sensors with a predictive solution was confirmed.	Rudack et al. [68]
	Monitoring the HPDC process and early defect detection based on sensor data	This research focused on developing a remote monitoring system for temperature and pressure in the HPDC mould cavity using thermocouple sensors. Measurements were taken at several points in the mould, and their reliability was verified using numerical simulations. The ability to observe local changes in process conditions and to use the data to identify potential casting defects was demonstrated. The results confirmed the effectiveness in improving the monitoring and control of the HPDC process.	Lee et al. [69]

**Table 3:** KPIs for monitoring the quality, microstructure, and properties of castings in HPDC core processes.

Main Process Stage	KPI	Template	Unit
Melting the alloy	Liquid metal temperature stability	${PG}_{1}=\frac{\sigma T}{\bar{T}}\cdot 100\%$ where σT—standard deviation of the liquid metal temperature; $\bar{T}$—average temperature of the molten metal	%
Cleaning and refining the molten metal	Gas porosity level	${PG}_{2}=\frac{V_{por}}{V_{próbki}}\cdot 100\%$ where *V_por_*—gas pore volume; *V_próbki_*—volume of the sample being tested	%
Filling the die-casting chamber	Filling volume repeatability	${PG}_{3}=\frac{\sigma_{V}}{\bar{V}}\cdot 100\%$ where $\sigma_{V}$—standard deviation of metal volume; $\bar{V}$—volume of the sample being tested	%
Phase I of the piston stroke	Phase I velocity stability	${PG}_{4}=\frac{\sigma_{V1}}{\bar{V_{1}}}\cdot 100\%$ where $\sigma_{V1}$—standard deviation of phase I velocity; $\bar{V_{1}}$—average speed of phase I	%
Phase II of the piston stroke	Underfill rate	${PG}_{5}=\frac{n_{ndl}}{\bar{n_{cał}}}\cdot 100\%$ where *n_ndl_*—number of underfilled castings; $\bar{n_{cał}}$—total number of castings	%
Pressure measurement	Shrinkage porosity reduction	${PG}_{6}=\frac{P_{bez}-\;P_{po}}{P_{bez}}\cdot 100\%$ where *P_bez_*—porosity level prior to intensification; *P_po_*—porosity level after intensification	%
Solidification of the casting	Average Secondary Dendrite Arm Spacing (SDAS)	${PG}_{7}=\frac{\sum {SDAS}_{i}}{n}$ where *SDAS_i_*—the local value of the distance between the secondary branches of dendrites measured in a given area of the sample; *n*—number of measurements taken	μm
Opening the mould and removing the casting	OEE (Overall Equipment Effectiveness) of HPDC machines	${PG}_{8}=A\cdot P\cdot Q$ where *A*—availability; *P*—performance; *Q*—quality	%
Removing the gating system	Material utilisation rate	${PG}_{9}=\frac{m_{wyr}}{m_{ws}}\cdot 100\%$ where *m_wyr_*—weight of the finished casting; *m_ws_*—total batch weight	%
Quality control	Scrap rate	${PG}_{10}=\frac{n_{br}}{n_{cał}}\cdot 100\%$ where *n_br_*—number of shortages; *n*_*ca*ł_—total number of castings produced	%

**Table 4:** Objectives and significance of key performance indicators for core processes in evaluating the quality, microstructure, and properties of aluminium alloys.

Main Process Stage	KPI	KPI Objective	Impact on Alloy Quality, Microstructure, and Properties
Melting the alloy	Liquid metal temperature stability	Ensuring stable thermal conditions for the metal melting process	Temperature stability affects the uniformity of chemical composition, minimises oxidation, and ensures microstructural reproducibility
Cleaning and refining the molten metal	Gas porosity level	Reducing gas content and improving the metallurgical quality of the alloy	This parameter determines the metallurgical quality of the alloy and directly influences the tightness and mechanical properties of castings
Filling the die-casting chamber	Filling volume repeatability	Ensuring the repeatability and stability of liquid metal dosing	The stability of molten metal feeding affects process repeatability and the reduction in volumetric defects
Phase I of the piston stroke	Phase I velocity stability	Reducing turbulence in the metal flow during the initial phase of the piston’s movement	Reducing flow turbulence minimises the risk of entrapment and the formation of gas defects
Phase II of the piston stroke	Underfill rate	Ensuring that the mould cavity is filled correctly during the second phase of the piston stroke	Underfilling indicates improper mould filling and may affect the structural integrity of the product
Pressure measurement	Shrinkage porosity reduction	Reduction in shrinkage defects (shrinkage porosity) and improvement in structural density	The indicator assesses the effectiveness of compacting the casting structure and reducing shrinkage defects
Solidification of the casting	Average Secondary Dendrite Arm Spacing (SDAS)	Controlling grain size and microstructural homogeneity	A parameter directly related to the alloy microstructure and the mechanical properties of castings
Opening the mould and removing the casting	OEE (Overall Equipment Effectiveness) of HPDC machines	Increasing the stability and efficiency of the production process	Comprehensive assessment of the stability of the production process and its impact on casting quality
Removing the gating system	Material utilisation rate	Reducing material losses and improving alloy utilisation	The indicator determines the efficiency of material utilisation and the level of aluminium alloy loss
Quality control	Scrap rate	Reducing the number of production defects	A key indicator of the final quality of the casting process and the stability of technological parameters

**Table 5:** KPIs for monitoring the quality, microstructure, and properties of castings in HPDC auxiliary processes.

Auxiliary Process Stage	KPI	Template	Unit
Charging preparation	Stability of the content of the controlled alloying element	${PP}_{1}=\frac{\sigma_{c}}{\bar{C}}\cdot 100\%$ where $\sigma_{c}$—standard deviation of the concentration of the element being analysed; $\bar{C}$—average alloy content $\sigma_{C}=\sqrt{\frac{1}{n-1}\sum_{i=1}^{n}{\left(C_{i}-\bar{C}\right)}^{2}}$ where *C_i_*—the content of the alloying element under analysis in the i-th sample [%]; $\bar{C}$—average content of the element analysed [%]; *n*—the number of chemical analyses carried out; $\sigma_{C}$—the standard deviation of the concentration of the element being analysed	%
Liquid metal transport	Temperature drop of molten metal during transport	${PP}_{2}=\;T_{strat}-\;{T\;}_{koniec}$ where *T_strat_*—metal temperature prior to transport; *T_koniec_*—metal temperature after transport	°C
Mould lubrication and preparation	Uniformity of the release agent layer	${PP}_{3}=\frac{\sigma_{L}}{\bar{L}}\cdot 100\%$ where $\sigma_{L}$—standard deviation of lubricant film thickness; $\bar{L}$—average layer thickness	%
Recycling of return material	Alloy recycling efficiency index	${PP}_{4}=\frac{m_{wtór}}{m_{cał}}\cdot 100\%$ where $m_{wtór}$—weight of recycled material used in the batch; $m_{cał}$—total batch weight	%

**Table 6:** Detailed interpretation of KPIs used to monitor the quality, microstructure, and properties of castings in HPDC auxiliary processes.

Process Stage	KPI	KPI Objective	Impact on Alloy Quality, Microstructure, and Properties
Charging	Stability of the chemical composition of the charge	Ensuring the chemical homogeneity of the alloy prior to melting	The stability of the chemical composition affects the reproducibility of mechanical properties and the uniformity of the microstructure
Transport of molten metal	Temperature drop of the molten metal during transport	Minimising heat loss during the transport of molten alloy	Excessive temperature drop affects alloy viscosity, mould filling conditions, and the microstructure of the casting
Mould lubrication and preparation	Uniformity of the release agent layer	Ensuring stable mould surface conditions	Lack of layer uniformity affects surface quality, local cooling conditions, and surface defects
Recycling of scrap material	Alloy recycling efficiency index	Evaluating the effectiveness of using recycled material without compromising alloy quality	An excessive proportion of scrap can lead to increased impurities and variability in the alloy’s microstructure

**Table 7:** Aligning KPIs for the high-pressure die-casting of aluminium alloys with Industry 4.0 objectives.

Process Stage	KPI	Data Sources	Connection to Industry 4.0
Charging	Stability of the chemical composition of the charge	Chemical composition analysers, laboratory (spectrometry), raw material supplier data	Digital chemical composition analysis, integration of laboratory data with MES/ERP, material batch traceability
Alloy melting	Stability of the molten metal temperature	Thermocouples, pyrometers, furnace temperature sensors	Real-time IoT monitoring, data archiving, thermal trend analysis
Purification and refining of molten metal	Level of gas porosity	Quality control systems, X-ray/CT inspections, laboratory test benches	Integration of quality data (CT/X-ray) with production databases, big data analytics
Transport of molten metal	Temperature drop during transport	Vat temperature sensors, data loggers	IoT monitoring, real-time heat loss analysis
Lubrication and mould preparation	Homogeneity of the release layer	Flow sensors, dosing systems, inspection cameras	Dosing automation, mould preparation parameter monitoring
Filling the pressing chamber	Repeatability of filling volume	Flow sensors, injection control system, PLC	Closed-loop control, process parameter logging
Phase I of piston movement	Stability of Phase I speed	Position sensors, encoders, machine control system	HPDC machine operation monitoring, process deviation analysis
Phase II of piston movement	Underfill rate	Data from the HPDC machine, process control systems, quality data	Machine learning, casting defect prediction, process data analysis
Pressure intensification	Reduction in shrinkage porosity	Pressure sensors, PLC loggers, control data	Adaptive control, real-time parameter analysis
Solidification of the casting	Average SDAS value	Microstructure analysis systems, digital microscopes, sample images	AI image analysis, microstructure databases
Opening the mould and removing the casting	HPDC machine OEE	MES system, machine data, cycle times	KPI dashboards, MES–SCADA–ERP integration
Removal of the gating system	Material utilisation rate	Weighing systems, production data, material records	Material flow monitoring, alloy utilisation efficiency analysis
Quality control	Scrap rate	Vision systems, quality reports, laboratory data	AI vision systems, big data analytics
Recycling of scrap material	Alloy recycling efficiency index	Scrap weighing systems, warehouse data, MES	Recycled material content monitoring, traceability, recycled material quality analysis

## Data Availability

The original contributions presented in this study are included in the article. Further inquiries can be directed to the corresponding author.

## References

[1] Luo T.Y., Qu J.J., Cheng S. (2025). Digital transformation, dynamic capability and total factor productivity of manufacturing enterprises. Ind. Manag. Data Syst..

[2] Tu J., Wei X., Bin Razik M.A. (2025). The impact of digital technology on total factor productivity in manufacturing enterprises. Sci. Rep..

[3] Miskiewicz R., Wolniak R. (2020). Practical Application of the Industry 4.0 Concept in a Steel Company. Sustainability.

[4] Gajdzik B., Grabowska S., Saniuk S., Wieczorek T. (2020). Sustainable development and Industry 4.0: A bibliometric analysis identifying key scientific problems of the sustainable Industry 4.0. Energies.

[5] Pacana A., Czerwińska K. Analysis of non-compliance for the cast of the industrial robot basis. Proceedings of the 28th International Conference on Metallurgy and Materials (Metal 2019).

[6] Liu X., Zhong S.B., Su Z., Zhou Y.R. (2025). The impact of digital transformation on high-quality development of manufacturing enterprises: An integrated perspective on efficiency and social responsibility. Mathematics.

[7] Shang M., Jia C.J., Zhong L.L., Cao J.W. (2024). What determines the performance of digital transformation in manufacturing enterprises? A study on the linkage effects based on fs/QCA method. J. Clean. Prod..

[8] Ingaldi M., Ulewicz R. (2025). Sustainable development and technological advancements in Industry 4.0: Overcoming barriers in SME sector integration. Manag. Syst. Prod. Eng..

[9] Kannan N.S., Parameshwaran R., Saravanakumar P.T., Kumar P.M., Rinawa M.L. (2022). Performance and quality improvement in a foundry industry using fuzzy MCDM and lean methods. Arab. J. Sci. Eng..

[10] Shetty R., Al Majali A., Wells L. (2026). Integrating spatial statistics and digital processing for enhanced surface quality classification in the foundry industry. Int. J. Met..

[11] Pacana A., Czerwińska K. (2020). Improving the quality level in the automotive industry. Prod. Eng. Arch..

[12] Guo Y.T., Yang J.Y., Yang C. (2026). A Review of applications and challenges of large language models for foundry intelligence in the casting industry. CMC-Comput. Mater. Contin..

[13] Ruberti M. (2024). Environmental performance and trends of the world’s semiconductor foundry industry. J. Ind. Ecol..

[14] Ulewicz R. (2003). Quality control system in production of the castings from spheroid cast iron. Metalurgija.

[15] Pacana A., Czerwińska K. (2023). Indicator analysis of the technological position of a manufacturing company. Prod. Eng. Arch..

[16] Zheng J., Chen A.K., Yao J.K., Ren Y.C., Zheng W., Lin F., Shi J.J., Guan A.Z., Wang W. (2022). Combination method of multiple molding technologies for reducing energy and carbon emission in the foundry industry. Sustain. Mater. Technol..

[17] Yi W., Liu G.C., Gap J.B., Zhang L.J. (2021). Boosting for concept design of casting aluminum alloys driven by combining computational thermodynamics and machine learning techniques. J. Mater. Inform..

[18] Sigworth G. (2011). Understanding quality in aluminum castings. Int. J. Met..

[19] Li D.Z., Slater C., Cai H.S., Hou X.A., Li Y.B., Wang Q.D. (2023). Joining technologies for aluminium castings—A review. Coating.

[20] Bruna M., Galcik M. (2021). Casting quality improvement by gating system optimization. Arch. Foundry Eng..

[21] Pacana A., Czerwińska K. (2021). Model of diagnosing and searching for incompatibilities in aluminium castings. Materials.

[22] Yu G.Q., Tang X.F., Deng L., Jin J.S., Gong P., Zhang M., Wang X.Y., Li Y.Y. (2026). A general data-driven framework for fully convex anisotropic yield criterion modeling. Int. J. Plast..

[23] Djurdjevic M.B., Odanovic Z., Pavlovic-Krstic J. (2010). Melt quality control at aluminum casting plants. Metall. Mate-Rials Eng..

[24] Lin R., Liu B., Zhang J.J., Zhang S.E. (2022). Microstructure evolution and properties of 7075 aluminum alloy recycled from scrap aircraft aluminum alloys. J. Mater. Res. Technol..

[25] Chen D.Z., Xu C., Yu J.Y., Wang Q., Fang H.Z., Yin S., Lookman T., Chen R.R. (2026). Interpretable Machine Learning Framework for Nb―Si based alloy design with enhanced fracture toughness. Adv. Sci..

[26] Wang Q.g., Wang J.F., Coryell J., Apelian D. (2025). Sustainable pathways to produce aluminum structural castings. J. Sustain. Metall..

[27] Pacana A., Pasternak-Malicka M., Zawada M., Radon-Cholewa A. (2016). Decision support in the production of packaging films by cost-quality analysis. Przem. Chem..

[28] Matejka M., Bolibruchova D., Podprocka R. (2021). A Study of Microstructure and Porosity Formation in High-Pressure Die-Casting. Arch. Foundry Eng..

[29] Koya E., Fukuda Y., Kitagawa S., Murakami M., Kawauchi A., Furue S. (2015). Manufacturing Technology for Hollow Structure Large Aluminum Parts Production by High Pressure Die Casting (HPDC). SAE Int. J. Passeng. Cars-Mech. Syst..

[30] Korti A.I.N., Abboudi S. (2017). Effects of shot sleeve filling on evolution of the free surface and solidification in the high-pressure die casting machine. Int. J. Met..

[31] Trometer N., Godlewski L.A., Prabhu E., Schopen M., Luo A.A. (2024). Effect of vacuum on die filling in high pressure die casting: Water analog, process simulation and casting validation. Int. J. Met..

[32] Majernik J., Podaril M., Majernikova M., Sramhauser K. (2025). Temperature conditions change in the high pressure die casting mold volume depending on the gating system volume. Arch. Foundry Eng..

[33] Chang E.C., Wang Q.G., Dharmavarapu B.R., Hu C. (2021). A novel durability analysis approach for high-pressure die cast aluminum engine block. SAE Int. J. Engines.

[34] Kang H.J., Yoon P.H., Lee G.H., Park J.Y. (2025). Effects of porosity on mechanical properties of castings in high-pressure die-casting process. Int. J. Met..

[35] Yang Q., Wu X.H., Qiu X. (2023). Microstructural characteristics of high-pressure die casting with high strength-ductility synergy properties: A review. Materials.

[36] Zhao H.D., Wang X.L., Wan Q., Bai W.H., Liu F. (2024). Characteristics and distribution of microstructures in high pressure die cast alloys with X-ray microtomography: A review. China Foundry.

[37] Adamane A.R., Arnberg L., Fiorese E., Timelli G., Bonollo F. (2015). Influence of injection parameters on the porosity and tensile properties of high-pressure die cast Al-Si alloys: A review. Int. J. Met..

[38] Niu G.D., Wang Y., Zhu L.J., Ye J.W., Mao J. (2022). Fluidity of casting Al-Si series alloys for automotive light-weighting: A systematic review. Mater. Sci. Technol..

[39] Xie H.C., Li Y.F., Song J.F., Hu H.R., He D.M., Li C.Y., Jiang B., Xiang D.X., Pan F.S. (2025). Effect of intensification casting pressure on microstructure and mechanical properties of high pressure die casting AE81 magnesium alloy. J. Mater. Res. Technol..

[40] Merchan M., Pascual A., Jimenez A., Garcia J.C., Anglada E., Galarraga H., Ortega N. (2025). High-pressure die casting (HPDC) process parameters optimization for Al-Mg-Fe aluminum alloy structural parts manufacturing. Metals.

[41] Arcaleni R., Girelli L., Tonelli L., Lattanzi L., Tocci M., Morri A., Pola A., Ceschini L. (2025). The role of high recycled content and heat treatments on microstructure, mechanical properties, and sustainability for an AlSi10MnMg structural automotive component. Sustain. Mater. Technol..

[42] Cantu-Fernandez D.S., Taha-Tijerina J.J., Gonzalez A., Hernandez P.G., Quinn B. (2024). Mechanical properties of a structural component processed in high-pressure die casting (HPDC) with a non-heat-treated aluminum alloy. Metals.

[43] Anand A., Nagarajan D., El Mansori M., Sivarupan T. (2023). Integration of additive fabrication with high-pressure die casting for quality structural castings of aluminium alloys; optimising energy consumption. Trans. Indian Inst. Met..

[44] Stomme E.T., Henderson H.B., Sims Z.C., Kesler M.S., Weiss D., Ott R.T., Meng F.Q., Kassoumeh S., Evangelista J., Begley G. (2018). Ageless aluminum-cerium-based alloys in high-volume die casting for improved energy efficiency. JOM.

[45] Dittrich D., Lehmhus D., Haesche M., Gomes L.F., Pille C., Jahn A., Ullmann L., Graner C. (2026). Influence of secondary aluminum content on casting and weldability of high pressure die cast materials for sustainable automotive body concepts. J. Adv. Join. Process..

[46] Miao Y.S., Li Z.Y., Tian Y., Hou Q.H., Wang S.H., Wang J.S. (2025). Progress in integrated die-casting technology for aluminum alloys. Int. J. Met..

[47] Pacana A., Czerwińska K. (2020). Comparative Tests of the Quality of the Piston Combustion Chamber for a Diesel Engine. Teh. Vjesn.-Tech. Gaz..

[48] Plentz P.D.M., Ribeiro T.M.M., Rahmig H.F.C., Bulcao-Neto R.F., Sene I.G., Morales H.F.C., Bulcao-Neto R.F., Sene I.G., Morales A.S. Digital transformation: Challenges about IoT and AI in High Pressure Die Casting (HPDC). Proceedings of the 2025 IEEE 19th International Symposium on Applied Computational Intelligence and Informatics (SACI).

[49] Began B., Baker E., Johanson J. (2026). Implementing an Industry 4.0 style degassing procedure into a V-process foundry making A356 aluminum alloy castings. Int. J. Met..

[50] Teamah A.M., Teamah A.M., Hamed M.S., Shankar S. (2025). Experimental and numerical replication of thermal conditions in high-pressure die-casting process. Processes.

[51] Chengjun W., Hao D., Long L. (2024). Design, simulation, control of a hybrid pouring robot: Enhancing automation level in the foundry industry. Robotica.

[52] Dinesh M., Arvind C., Mole S.S.S., Kumar C.S.S., Sekar P.C., Somasundaram K., Srihari K., Chandragandhi S., Sundramurthy V.P. (2022). An energy efficient architecture for furnace monitor and control in foundry based on Industry 4.0 using IoT. Sci. Program..

[53] Disselhoff T., Martin R.J. (2026). Data-driven early quality prediction in high pressure die casting. Int. J. Met..

[54] Barot R.S., Patel H., Devmurari R., Shah K., Sharma B., Shah J. (2020). IoT feasibility aspects of mold temperature monitoring and casting simulation for smart foundry. Mater. Today-Proc..

[55] Arkhipov M.V., Matrosova V.V., Volnov I.N. (2020). Automation in foundry industry: Modern information and cyber-physical systems. Adv. Autom..

[56] Pacana A., Czerwińska K., Bednarova L., Simkova Z. (2025). Integration of key performance indicators (KPI) taxonomy and energy efficiency analysis in the aluminium industry using Industry 4.0 technologies. Energies.

[57] Perzyk M., Dybowski B., Kozlowski J. (2019). Introducing advanced data analytics in perspective of Industry 4.0 in a die casting foundry. Arch. Foundry Eng..

[58] Liszka K., Klimkiewicz K., Malinowski P. (2019). Polish foundry engineer with regard to changes carried by the Industry 4.0. Arch. Foundry Eng..

[59] Kang J.W., Liu B.l., Jing T., Shen H.F. (2024). Intelligent casting: Empowering the future foundry industry. China Foundry.

[60] Dinesh M., Arvind C., Srihari K. (2022). An efficient industry 4.0 architecture for energy conservation using an automatic machine monitor and control in the foundry. Automatika.

[61] Wu X.X., Du Y., Tian X.J., Liu D., Cong M. (2025). Multi-objective optimization of high-pressure die casting process parameters based on data-driven surrogate model. Int. J. Met..

[62] Niu Z.C., Liu G.Y., Li T., Ji S.X. (2022). Effect of high pressure die casting on the castability, defects and mechanical properties of aluminium alloys in extra-large thin-wall castings. J. Mater. Process. Technol..

[63] Koru M., Serce O. (2024). Experimental and theoretical investigation of heat transfer in vacuum assisted high pressure die casting (HPDC) process. Int. J. Met..

[64] Tariq A., Tariq A., Masud M., Rehman Z. (2022). Minimizing the casting defects in high-pressure die casting using Taguchi analysis. Sci. Iran..

[65] Dou K., Zhang Y.J., Lordan E., Jacot A., Fan Z.Y. (2022). Understanding the initial solidification behavior for Al-Si alloy in cold chamber high-pressure die casting (CC-HPDC) process combining experimental and modeling approach. Metall. Mater. Trans. A-Phys. Metall. Mater. Sci..

[66] Yang J., Liu B., Shu D.W., Li H.Z., Yang Q., Hu T.G., Wang Z.B., Zeng Y.B., Huang J., Tang X.L. (2025). Effect of ultra vacuum assisted high pressure die casting on the mechanical properties of Al-Si-Mn-Mg alloy. J. Alloys Compd..

[67] Dou K., Lordan E., Zhang Y.J., Jacot A., Fan Z.Y. (2020). A complete computer aided engineering (CAE) modelling and optimization of high pressure die casting (HPDC) process. J. Manuf. Process..

[68] Rudack M., Rom M., Bruckmeier L., Moser M., Pustal B., Buhrig-Polaczek A. (2024). Recurrent neural networks as virtual cavity pressure and temperature sensors in high-pressure die casting. Int. J. Adv. Manuf. Technol..

[69] Lee S., Han D., Kim N. (2025). Development and validation using casting simulation of a multi-point remote monitoring system for high-pressure die-casting (HPDC) mold cavity. Int. J. Met..

[70] Vanli A.S., Akdogan A., Kerber K., Ozbek S., Durakbasa M.N. (2018). Smart die casting foundry according to industrial revolution 4.0. Acta Tech. Napoc. Ser.-Appl. Math. Mech. Eng..

[71] Kucukaltan B., Irani Z., Aktas E. (2016). A decision support model for identification and prioritization of key performance indicators in the logistics industry. Comput. Hum. Behav..

[72] Lucianetti L., Battista V., Koufteros X. (2019). Comprehensive performance measurement systems design and organizational effectiveness. Int. J. Oper. Prod. Manag..

[73] Haraldsson J., Johnsson S., Thollander P., Wallen M. (2021). Taxonomy, saving potentials and key performance indicators for energy end-use and greenhouse gas emissions in the aluminium industry and aluminium casting foundries. Energies.

[74] Czerwińska K., Pacana A. (2022). Analysis of the maturity of process monitoring in manufacturing companies. Prod. Eng. Arch..

[75] Tu Y.M., Chen H.N., Chang S.H. (2010). The design of key performance indicators on technology development of a wafer foundry. J. Ind. Prod. Eng..

[76] Sherif Z., Sarfaz S., Jolly M., Salomitis K. (2022). Identification of the right environmental KPIs for manufacturing operations: Towards a continuous sustainability framework. Mater..

[77] Vijayanand J., Rao V.S., Karthikeyan K.M.B., Hemanandh J., Barmavatu P. (2025). An integrated approach to improving manufacturing KPIs using lean tools, multi-criteria decision-making, and neural network analysis. Int. J. Interact. Des. Manuf..

[78] Ulewicz R., Czerwińska K., Pacana A. (2023). A rank model of casting non-conformity detection methods in the context of Industry 4.0. Materials.

[79] Balon U., Dziadkowiec J.M., Niewczas-Doborowalska M. (2024). Key performance indicators (KPIs) in the quality management system. Int. J. Qual. Res..

[80] Oliveira M.R., Jorge D., Pecas P. (2019). Methodology of operationalization of KPIs for shop-floor. Handbook of Research on Green Engineering Techniques for Modern Manufacturing.

